# Consumer Group Identification Algorithm for Ice and Snow Sports

**DOI:** 10.1155/2022/2174910

**Published:** 2022-06-29

**Authors:** Ting Zhang, Wei Wang

**Affiliations:** ^1^Department of Physical Education, Zhongnan University of Economics and Law, Wuhan 430073, Hubei, China; ^2^Sports Training Institute, Wuhan Sports University, Wuhan 430079, Hubei, China

## Abstract

As an important part of the modern sports industry system, the quality and level of its development are related to whether China's sports industry can successfully become a pillar industry of the national economy. Therefore, the development of the ice and snow sports industry is to promote the expansion of China's sports industry scale high quality development of the national economy and an important way to build sports power. Participative sports consumption is the most important part of sports consumption and the development of the sports industry. The sports industry separated from participative sports consumption is water without source and tree without roots, while participative sports consumption demand is the power source of participative sports consumption. At present, there is no systematic and complete research on participation sports consumption demand. In order to understand the causes and demand state of residents' participation sports consumption demand and provide entry points for enterprises to formulate marketing strategies, this study constructs an organic system with participation sports service products as consumption objects, centering on the demanding state of participation sports consumers. In the system, on the theory of supply and demand, under the guidance of consumption economics theory, adhere to the combination of theoretical research and empirical analysis, the combination of macroplanning and microdesign, the combination of qualitative analysis and quantitative analysis, through the empirical investigation and receipt collection of residents' participation sports consumption demand, the use of systematic analysis, literature method, and survey method, through mathematical analysis, and other research methods, the paper explores the main causes and demand conditions of residents' participation sports consumption demand in different consumption states and excavates the main causes and demand conditions of participating sports consumption demand in different consumption states under different sports levels.

## 1. Introduction

At present, China is in the period of social transformation, increasingly showing the characteristics of a consumer society, traditional economic development is facing a test, and many industries have encountered bottlenecks. In sharp contrast, the sports industry has maintained a relatively high growth rate in recent years, increasingly showing a trend of explosive development. The sports consumption market has increasingly become a new blue ocean consumer society to stimulate demand and promote economic growth [1]. With the advent of sports industry development, the dual background requires more attention to the study of sports consumption. Under the new social background, sports consumption shows new characteristics and phenomena, calling for a new research perspective. In the big family of consumption, sports consumption develops rapidly and has great potential. Since the reform and opening up, the consumption concept of Chinese residents has undergone great changes, and the consumption structure has been constantly improved. As the sports consumption to meet the needs of development and enjoyment, it has been paid more and more attention. In recent years, rapid growth in China's sports consumer groups, the rise of social leisure sports is to develop sports industry as the most promising sunshine industry, on the basis of sports consumption of the sports industry has become a new economic growth point, and sports consumption in the national economy has an important position and is in the process of rapid development [2, 3].

The ice and snow sports industry is an important component of the tertiary industry. Developing the ice and snow sports industry is one of the main ways to promote the high-quality development of China's sports industry, and also a key factor to promote the upgrading of sports consumption and industrial development. Therefore, it has become an important guiding scheme to promote the structural transformation of China's sports industry to explore the development of the ice and snow sports industry and deeply explore the direction of industrial consumption upgrading and supply transformation. In addition, scientific research and discussion have important academic value and practical significance for discovering the relationship between the forms of the ice and snow sports industry, improving the chain of the ice and snow sports industry, strengthening the service improvement of the ice and snow sports industry [4], solving the dilemma of sports industrial structure transformation, and promoting the coordinated development of sports economy [2, 5].

For the development strategy of the ice and snow sports industry in China, this to improve the internal structure of the ice and snow sports industry in China has a certain practical application value visible, study the development strategy of the ice and snow sports industry in China, is mainly embodied in the following points [6–8]: first, the structure transformation of the ice and snow sports industry in China is dependent on the policy for the development of the ice and snow sports industry in our country, and investment driven development mode innovation and regional economic driver and so on all has the vital significance. Second, the practice shows that the development of the ice and snow sports industry process will affect the linkage mechanism between government departments, and microlevel research can to a certain extent. Guidance on the ice and snow sports industry economic reengineering practice process such as overflow and value. Third, in much starker choices and graver consequences in sport industry development goals more clearly, mining consumption patterns. To explore psychological and emotional distinguish between consumer groups such as microresearch to guide the high quality of the ice and snow sports industry development in our country has important academic value.

In addition, under the background of the new era, the ice and snow sports industry has become a new impetus for the construction of China's economic power, which is conducive to the transformation of economic development mode and adjustment of industrial structure. Fourth, from the perspective of regional economic development, the ice and snow sports industry are affected by geographical environment and has distinct regional characteristics [9, 10]. Therefore, the study of the ice and snow sports industry can promote the development of the regional economy, which has important theoretical significance for improving the comprehensive influence of cities and helping the construction of economic power. In conclusion, based on the reflection of academic research, the research on the ice and snow sports industry can form huge market value and its solid theory guides and promotes the sustainable development of the ice and snow sports industry [11–13].

**Figure 1:**
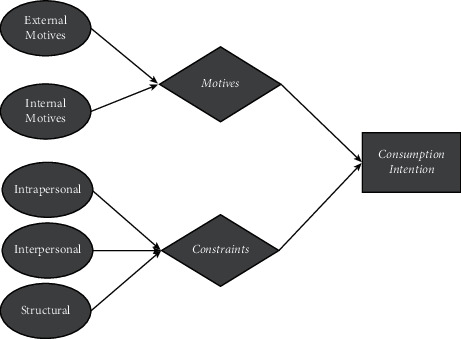
Factors influencing consumption intention of ice and snow sports.

As shown in Figure 1, the factors influencing consumption intention of ice and snow sports are given. Behind the vigorous development of the ice and snow sports industry, there is always the problem of insufficient discussion among the consumer groups of ice and snow sports, and behind the growth of the macroeconomy, there is also the problem of the weak contribution of microbehavior research to the promotion and industrial development [14, 15]. At the same time, identifying consumer groups for ice and snow sports operation not only needs the support of management theory, behavioral theory, and public management theory but also needs to conduct in-depth discussion on consumer behavior issues related to the microbehavioral field, which is the motivation of this research topic [16–18]. However, due to the current existing in the research of the development of the ice and snow sports industry most focused on the games theory, ice and snow sports economy and policy guidance, etc., lack of consumer demand, word-of-mouth based microscopic behavior such as consumer behavior research, which not only cause the lack of corresponding microscopic behavior in theoretical research. At the practical level, many demand-side problems in the development of the ice and snow sports industry have not been explored and solved. For example, why ice and snow sports products can stimulate consumers to consume ice and snow sports? Why can the behavioral needs of consumers affect the transformation of the ice and snow sports industry? Why consumers' word-of-mouth information can affect product sales and the development of the whole industry? The above-given questions are the profound reflection of microbehavior in the perspective of management of the ice and snow sports industry [19].

The consumption behavior management in this study mainly focuses on the exploration of microconsumption behaviors in the ice and snow sports industry; that is, the study mainly discusses the influence of relevant consumer demand-side factors on the development of the ice and snow sports industry from the perspective of consumers [20–22]. In order to determine which relevant consumer demand-side factors have the most obvious or meaningful impact on the development of the ice and snow sports industry and to attract the attention of relevant managers of the ice and snow sports industry in the subsequent practice and development process, this study is based on the attribution theory. Based on grounded theory, it is concluded that consumer demand and word-of-mouth communication are two key factors affecting the development of ice and snow sports industry at the microbehavioral level; that is, in the basic link of the development of ice and snow sports industry, there should be effective interaction between consumer demand and word-of-mouth communication to jointly promote the development of the industry. Through literature review, it is found that some qualitative studies show that consumer demand can promote the improvement of products, services, consumption, and other industrial contents of the ice and snow sports industry, promote the coordinated development of the ice and snow sports industry chain, and drive the overall development of the ice and snow sports industry [23]. Meanwhile, word of mouth, as consumers' opinions on products or services in the process of consumption, can affect the supply quality, service level and industrial structure of the ice and snow sports industry, enhance the development competitiveness of the ice and snow sports industry, and ensure the sustainable development of the ice and snow sports industry [24, 25].

The reminder of this paper is outlined as follows: Section 2 gives the related work, then the consumer group identification and analysis for ice and snow sports is introduced in Section 3. After that, Section 4 shows the experimental results and analysis. Finally, Section 5 concludes the paper.

## 2. Related Work

In recent years, various fields in China have begun to apply grounded theory to the process of attribution, and some achievements have been achieved. In related studies in the field of education, Gong et al. [26] studied the phenomenon of attention-loss in the fragmented learning process of college students through the qualitative method of grounded theory. Lack of learning input and lack of internal drive are the fundamental factors that affect the attention of college students. In communication studies, Yeh et al. [27] using the grounded theory by Dolce & Gabbana incident cases of humiliating to China, summed up China's netizens mood spread of form elements of the construction of the network public opinion transmission model of emotional via the method of grounded theory of qualitative implementation problems of grind attribution is a feasible research train of thought, is root. The process of attribution is an effective qualitative method to find out influencing factors for the study of problems. The combination of grounding and attribution is of great significance to improve the accuracy and scientificity of social science research. Lei et al. [28] believed that China's ice and snow sports industry should choose different modes according to local conditions. The development of enterprise mode should be strengthened in golden areas with strong foundation and strong potential; the mixed mode of joint development between government and enterprises can be adopted in areas with strong foundation or potential; and the community mode can be adopted in areas with weak foundation and weak potential. Naraine et al. [29] believed that sports consumption contains three meanings: first, the subject of sports consumption is consumers with consumption ability. Second, to meet the enjoyment and development needs of consumers; third, the consumer (subject) to the consumption of materials and labor (object) of the dependent relationship.

The second is to acquire the right to rebroadcast the combined marketing events by buying the right to rebroadcast sports events; The third is to acquire franchise rights of sports organizations or sports events through payment. Abdolmaleki and Babaei [30] collected and analyzed a large number of research data on stadium operation mode. It is pointed out that large stadiums and gymnasiums can promote local economic development to a certain extent but also have some negative effects. Hautbois et al. [31] believed that the PPP financing mode adopted by some stadiums and gymnasiums in the United States can provide a good reference for the operation and tax measures of stadiums and gymnasiums. Cho et al. [32] take adolescent girls' smoking behavior as an example to discuss the relationship between leisure conspicuous consumption and identity. Referring to Veblen's theory, they believe that the surge in adolescent girls' smoking in the 1990s is because they try to acquire a specific identity through smoking as a leisure behavior. Physical activities such as tennis, cycling, surfing, dancing, and outdoor sports are more healthy and socially valuable than mere material conspicuous consumption by demonstrating a healthy and fashionable way of leisure.

In western developed countries, the core functions of stadiums and gymnasiums are to provide fitness facilities and organize community fitness services, spread sportsmanship and culture, monitor mass sports activities, and organize competitive sports. European stadiums mainly rely on their own operation to complete commercial operations, through the organization of clubs to rent stadiums and member discounts. The operation funds obtained through channels, partly funded by the government, can not only meet the personal needs of others for fitness training but also carry out marketing activities and sports events to improve the operation efficiency of the stadium and develop different business strategies to promote long-term development. Cuesta-Valiño et al. [33] driving a luxury car to play golf in Las Vegas and other places as a typical conspicuous consumption activity. High-end sports clubs have become the new favorite of conspicuous consumption. In the United States, as a symbol of social status, joining expensive clubs has become very popular among young people. The rich can also be seen in top private clubs, but membership is by invitation only. This logical thinking is concise and clear, which intuitively reflects the close correlation between conspicuous consumption of sports and its social value. However, simple linear relationship cannot completely restore the complex relationship between these variables. Rana and Mittal [34] reviewed the history of the design of football sneakers in the UK and other countries; from the initial competition equipment of a few athletes to later slowly out of the stadium, football shoes have become a fashion and leisure choice and gradually become a chip for people to achieve conspicuous consumption, to show their identity, wealth, taste, and social division. Since the middle of the 20th century, the brand's pursuit of star effect has become the new favorite for people to achieve social location separation. The influence of mass media on manufacturers is more and more significant, and manufacturers pay more and more attention to advertising. This process also happened in the development of sports equipment such as tennis and golf.

Through literature review, the research on sports consumption and sports demand has a further understanding. The research content of sports consumption demand is closely linked with the development of sports industry and the development level of sports consumption market due to the high level of sports industry and sports consumption in developed countries, the market development is more perfect and the research content and research level are correspondingly more mature and in-depth. Second, many scholars pay more attention to applied research, highlighting the service for the market. The research methods are mainly quantitative empirical analysis. The research ideas are mostly problem-oriented. Most of the research theories are supported by marketing, management, and economic theories. Third, the research studies of scholars focus more on theoretical analysis, and the research content mostly focuses on the current characteristics and countermeasures of sports consumption. Qualitative analysis is the main research method and quantitative analysis is increasing; the theoretical support of the research is relatively weak and lacking and lacks of strong and in-depth theoretical analysis. First, as we all know, sports consumption in the narrow sense usually refers to participatory sports consumption, and sports consumption in the broad sense includes participatory sports consumption, paraphernal-type sports consumption, ornamental sports consumption, and so on. It can be seen that participatory sports consumption is the core of sports consumption, and both paraphernal-type sports consumption and ornamental sports consumption should be attached to the participation. And, the type of sports consumption and the development and prosperity, from participating sports consumption and sports consumption is difficult to grow. Nowadays, the study of generalized sports consumption in China is more, and the researchers are mostly in the process of generalized sports consumption is discussed, mention the participative sports consumption, the participative sports consumption macroscopic, this paper expounds the structure status. The research results are of low reference value in practice and lack of attention to participatory sports consumption. There are only a few studies on participatory sports consumption, so it is urgent to pay attention to and explore potential.

## 3. Consumer Group Identification and Analysis for Ice and Snow Sports

### 3.1. The Flowchart of the Identification Method

**Figure 2:**
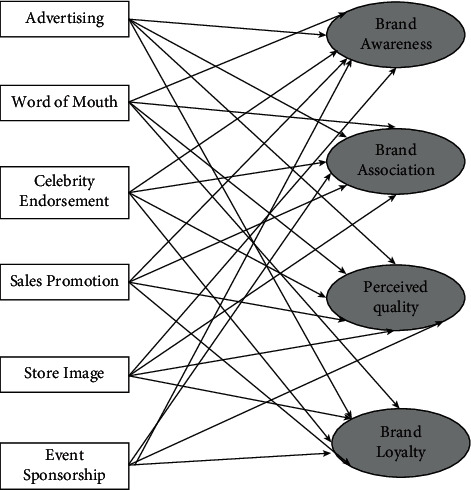
Flow chart of ice and snow sports consumer group identification.

After three steps of rooted coding, this paper clarified and clarified the core category of sports venues consumption behavior influence factors and main category psychological factors social appeal internal operation external environment sociology demographic variables and sorted out the relationship structure between the main categories, on this basis, combined with the two-channel psychology. The concept of account, this paper constructs a large public sports venues consumption behavior influencing factors model. The whole system of the method is given in Figure 2. As can be seen from the figure, the direct correlation between different types of snow and ice sports and different consumer groups can be well characterized by the proposed method.

According to the building of large public sports venues factors affecting consumer behavior theory model, this paper puts forward the following four types of assumes that the interpretation of the sports consumption behavior influence mechanism, respectively, for the individual psychological factors on the relationship between the sports consumption behavior hypothesis relationship between.

Situational factors and sports consumption behavior hypothesis dual channel mental account adjustment hypothesis on the relationship between social demographic statistical variables and consumption behavior in sports venues: (1) the hypothesis of the influence of individual psychological factors on the consumption behavior of sports venues; (2) the hypothesis of the correlation between situational factors and sports consumption behavior; (3) hypothesis of the moderating effect of dual-channel mental accounts; (4) the hypothesis of the influence of social demographic statistical variables on the consumption behavior of sports venues.

### 3.2. Dynamic Equations of Evolutionary Game Models

The consumer benefit that the government chooses to implement the regulatory strategy is [14]

(1) $$\begin{array}{c} E_{G}^{Y}=yz\left(s-b-r\right)+y\left(1-z\right)\left(-r\right)+\left(1-y\right)z\left(-b-r+p-k\right)+\left(1-y\right)\left(1-z\right)\left(-r\right), \end{array}$$

where *E*_*G*_^*Y*^ is the expectation. The consumer benefit that the government chooses to implement the nonregulatory strategy is

(2) $$\begin{array}{c} E_{G}^{N}=yz\left(s-b\right)+\left(1-y\right)z\left(-b-k\right)+\left(1-y\right)\left(1-z\right)\left(-b\right), \end{array}$$

where *b* and *k* are the parameters. The government chooses the expected return of regulatory and nonregulatory strategies with probabilities of *X* and 1 − *x*.

(3) $$\begin{array}{c} E\left(G\right)=xE_{G}^{Y}+\left(1-x\right)E_{G}^{N}. \end{array}$$

Then, the replication dynamic equation of the government is

(4) $$\begin{array}{c} F\left(x\right)=\frac{dx}{dt}=x\left[E_{G}^{Y}-E\left(G\right)\right]=x\left[E_{G}^{Y}-xE_{G}^{Y}-\left(1-x\right)E_{G}^{N}\right]\\ =x\left(1-x\right)\left(E_{G}^{Y}-E_{G}^{N}\right)=x\left(x-1\right)\left(r-b+by+bz-pz-byz+pyz\right). \end{array}$$

The expected revenue of sports venues choosing ticket reduction or sports consumption coupons is

(5) $$\begin{array}{c} E_{S}^{Y}=0. \end{array}$$

The expected revenue of sports venues choosing not to reduce or exempt tickets or not to issue sports consumption vouchers is

(6) $$\begin{array}{c} E_{S}^{N}=xz\left(b-p\right)+\left(1-x\right)zb+\left(1-x\right)\left(1-z\right)b. \end{array}$$

The stadium chooses ticket reduction or sports consumption coupon with probability *y* and 1 − *y*, and the expected revenue without ticket reduction or sports consumption coupon is

(7) $$\begin{array}{c} E\left(S\right)=yE_{S}^{Y}+\left(1-y\right)E_{S}^{N}. \end{array}$$

Then, the replication dynamic equation of public stadiums is

(8) $$\begin{array}{c} F\left(y\right)=\frac{\text{d}y}{\text{d}t}=y\left[E_{S}^{Y}-E\left(S\right)\right]\\ =y\left[E_{S}^{Y}-yE_{S}^{Y}-\left(1-y\right)E_{S}^{N}\right]\\ =y\left(1-y\right)\left(E_{S}^{Y}-E_{S}^{N}\right)=y\left(y-1\right)\left(b-bx+bxz-pxz\right). \end{array}$$

The expected income of residents choosing to participate in exercise is

(9) $$\begin{array}{c} E_{P}^{Y}=xy\left(b-c\right)+x\left(1-y\right)\left(-c\right)+\left(1-x\right)y\left(b-c\right)+\left(1-x\right)\left(1-y\right)\left(-c\right), \end{array}$$

where *b* and *c* are the parameters. The expected income of residents who choose not to participate in exercise is

(10) $$\begin{array}{c} E_{P}^{N}=0. \end{array}$$

The expected benefits of residents choosing to participate in exercise and not participating in exercise with probability *z* and 1 − *z* are

(11) $$\begin{array}{c} E\left(P\right)=zE_{P}^{Y}+\left(1-z\right)E_{P}^{N}. \end{array}$$

Then, the replication dynamic equation of residents is

(12) $$\begin{array}{c} F\left(z\right)=\frac{\text{d}z}{\text{d}t}=z\left[E_{B}^{Y}-E\left(B\right)\right]=z\left[E_{B}^{Y}-zE_{B}^{Y}-\left(1-z\right)E_{B}^{N}\right]\\ =z\left(1-z\right)\left(E_{B}^{Y}-E_{B}^{N}\right)=z\left(z-1\right)\left(c-by\right). \end{array}$$

## 4. Experimental Results and Analysis

### 4.1. Introduction to Experimental Data Set

The previous section constructed a participatory sports consumption demand questionnaire and explained the item source and theoretical basis of each item. This section will use this questionnaire to test the reliability and validity of the questionnaire through presurvey item analysis, exploratory factor analysis, confirmatory factor analysis, and other factors and further simplify questions. The paper extracts common factors and establishes the index system of participatory sports consumption demand system.

The presurvey was conducted through street encounter and network survey (spread through circle of friends). A total of 787 questionnaires were issued, including 600 by street encounter, 187 by network survey, and 763 questionnaires were collected, including 575 by street encounter and 187 by network survey. In the questionnaire of street encounter, 6 questionnaires were invalid due to confusion. Meanwhile, 48 samples with identical choices in the questionnaire of street encounter and online survey were deleted, and 709 valid questionnaires were finally obtained, among which 111 were affected by not participating in physical exercise (hereinafter referred to as not exercising). 271 cases were affected for not participating in sports consumption (hereinafter referred to as “no consumption”), 147 cases were affected for not participating in sports consumption (hereinafter referred to as “no consumption”), and 180 cases were encouraged to participate in or continue to participate in sports consumption (hereinafter referred to as “continuous consumption”).

### 4.2. Experimental Results Analysis

**Figure 3:**
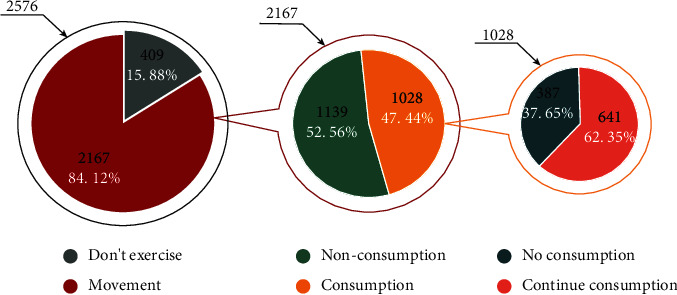
Citizens physical exercise, sports consumption structure distribution.

The physical exercise frequency characteristics of the total effective samples in this study are shown in Figure 3. There are 2576 effective samples, among which 2167 have physical exercise experience in the past 6 months, accounting for 84.12%. However, 409 people have no physical exercise experience in the past 6 months, accounting for 15.88%, which shows that the group that loves sports occupies the majority.

Among 2167 people who participated in physical exercise in the past 6 months, 1028 (47.44%) had participated in physical consumption. However, 1139 people have no experience of participating in sports consumption, accounting for 52.56%. It can be seen that among the people who participate in physical exercise, the level of participating in sports consumption is less than 50%, and more than half of the group do not consume, indicating a large stock of potential sports consumers.

Among the 1028 people who had participated in sports consumption, 641 people still engaged in sports consumption in the past 6 months, accounting for 62.35%; in the past 6 months, 387 people stopped participating in sports consumption, accounting for more than 1/3 of the total. It can be seen that in the samples with consumption experience, although people can continue to consume sports and their level is relatively good, the proportion of losing participating sports consumers is also worth paying attention to.

**Figure 4:**
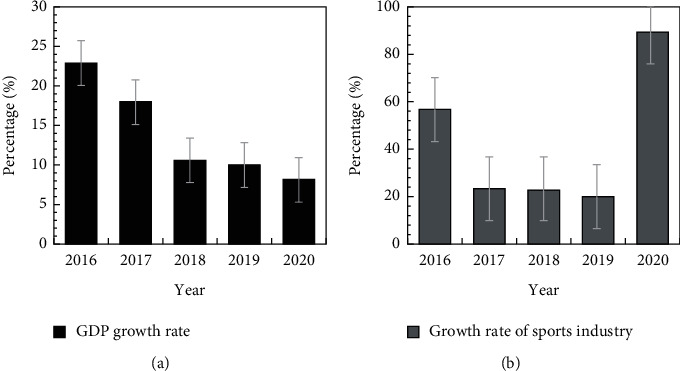
Comparison of added value: (a) GDP growth rate and (b) sports industry growth rate from 2016 to 2020.

Figure 4 gives the comparison of sports industry added value growth rate and GDP growth rate from 2016 to 2020. It can be seen from the figure that from 2016 to 2020, the GDP growth rate decreased year by year, and except for 2016, the size of the sports industry showed the increasing trend.

In order to explore the influence of the amount of financial subsidies issued by the government on the evolution path of the system, the amount of government financial subsidies was changed, respectively, to observe the changes of the system.

**Figure 5:**
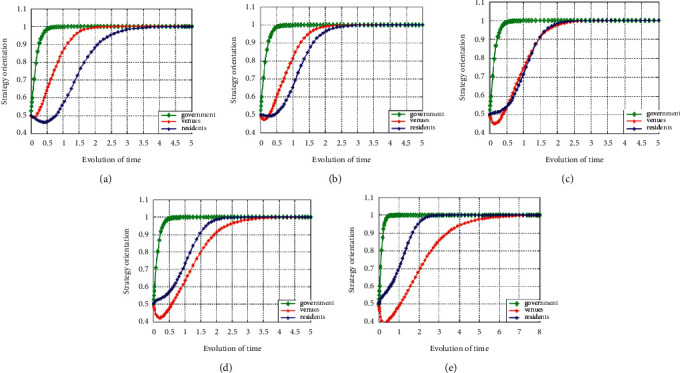
The evolution of government subsidy amount changes under (a) condition 1, (b) condition 2, (c) condition 3, (d) condition 4, and (e) condition 5.

It can be seen from Figure 5 that, with the increase of *B*, the time for the system to evolve to a stable state decreases first and then increases, indicating that there is a maximum amount of financial subsidy allocated by the government to stadiums; that is, it is greater than the total net cost of residents to exercise in stadiums. At the same time, it is also less than the total amount of penalties imposed by stadiums that do not reduce or exempt tickets according to regulations or do not issue sports consumption vouchers. The higher the financial subsidy allocated by the government to stadiums and gymnasiums, the better, because the subsidy is too low to make up for the cost loss of stadiums and gymnasiums, but too high will indirectly reduce the default cost of stadiums and gymnasiums, resulting in stadiums and gymnasiums would rather be punished for not reducing or reducing tickets or not issuing sports consumption vouchers according to regulations. They do not want to spend money and manpower to implement the open standards required by the state.

**Figure 6:**
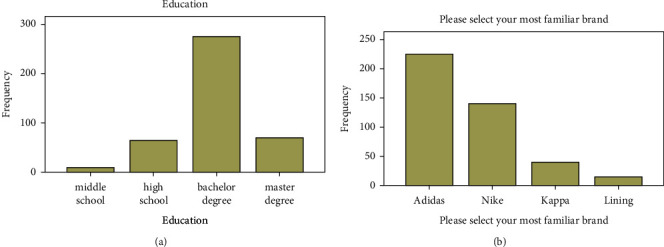
The relationship between sports consumption and (a) education level and (b) brand awareness.

Figure 6 shows the relationship between education level and brand awareness and sports consumption. People with undergraduate degrees and sports consumption frequency is the highest, people with a secondary school education and sports consumption frequency is the lowest. In addition, as can be seen from the chart below, Adidas is the most popular brand and the frequency of buying sports products of Li Ning is the lowest.

**Figure 7:**
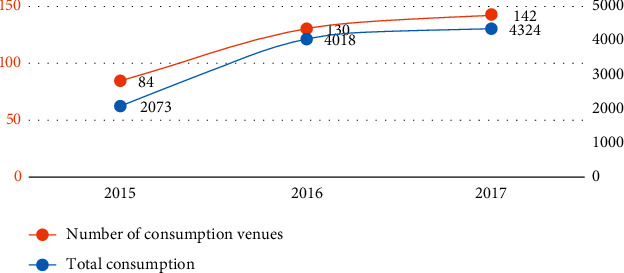
Chart of the number and total amount of financial subsidies for public (large) stadiums from 2015 to 2017.

Combination of sports venues in open work for free low-cost public sports service ability, service quality, and service level, the comprehensive performance evaluation results, using the Treasury's stadium free low-cost subsidies and incentives for capital and departments to award of Zhejiang province generation filling, the province related venues completed a total of 104.15 million yuan of financial aid. As can be seen from Figure 7, from 2015 to 2017, the total amount of financial subsidy funds for the free and low-charge opening of public (large) stadiums has been increasing, and both the number of subsidized stadiums and the total amount of subsidy have increased significantly.

**Figure 8:**
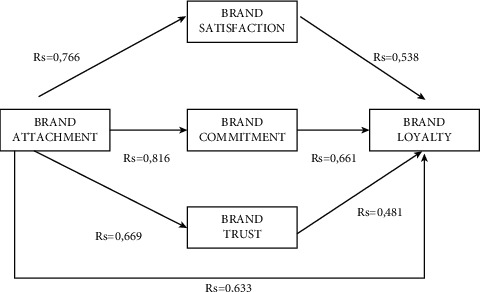
The influence of different factors on sports consumption weight coefficient.

In addition, the influence of different factors on sports consumption weight coefficient is given in Figure 8. It can be seen that different parameters have different impacts on operating consumer groups, and the difference is also large.

**Figure 9:**
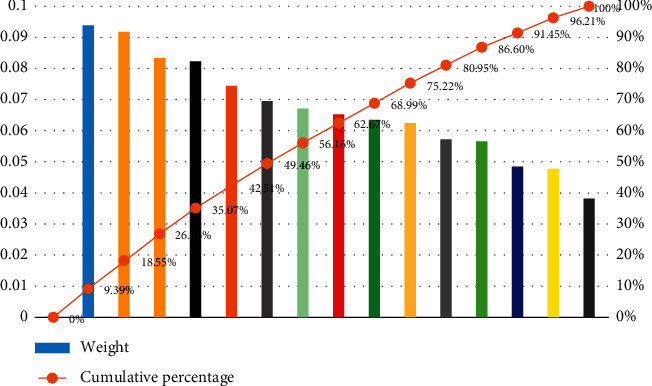
The sports complex service provider selects a Pareto diagram for measuring index weights.

As can be seen from Figure 9 that the cumulative percentage of indexes such as credit level, consumption-driven operation mode, content innovation, industry driving target market, industry affecting public image, and professional talents is 68.99%, approaching 70%, which has become an important indicator affecting the selection of sports complex service providers.

## 5. Conclusions

For the sportsman, the main influencing path of nonconsumption group is from consumption restriction to consumption preference and economic ability to low consumption preference, which is consistent with the influencing factors of the three movement levels, indicating that personal consumption habit has a huge impact on consumer behavior, while the main influencing path of nonconsumption group is from consumption cessation to consumption expectations and economic capacity to economic weakness. This is the most direct manifestation of the superstructure determined by economic basis on individuals. Economic ability determines a person's lifestyle and consumption style, which includes consumption habits of interests and hobbies. Compared with clothing, food, housing, and transportation, sports consumption, as the weakest link in life consumption, is most likely to be suspended. Finally, continued consumption group of body is the main effect of path consumption to promote to the external cause and economic ability. To the desired effect, this is the best proof of behavior results reflect behavior purpose, when the expected effect and the behavior of behavior purpose is consistent, both actions will continue at the same time also is the external cause through the explanation of its internal work, and anticipated effect is the external cause. And, consumption purpose is internal cause and continuous consumption is the most appropriate performance.

There are still some shortcomings in this paper: first, the results of this study show that the demographic characteristics of the samples are basically consistent with the overall demographic characteristics of Shanghai. However, due to the limitation of research funds, the sample collection is completed through the combination of network survey and interception survey, which is different from the completely random survey in terms of representativeness. In the process of analysis, the representative of the sample is fully considered, and corresponding supplementary work is carried out. In the future, when conditions are mature, data collection will be carried out in a completely random way.

## Data Availability

The data used to support the findings of this study are available from the corresponding author upon request.
